# Rare variability in adrenoleukodystrophy: a case report

**DOI:** 10.1186/s13256-018-1722-z

**Published:** 2018-06-28

**Authors:** Yanming Chen, Farhana Polara, Anjana Pillai

**Affiliations:** Department of Medicine, Lincoln Medical Center, 234 East 149th Street, Bronx, New York 10451 USA

**Keywords:** Adrenoleukodystrophy, Phenotypes, Adrenomyeloneuropathy, *ABCD1*

## Abstract

**Background:**

X-linked adrenoleukodystrophy is a genetic disorder with diverse clinical phenotypes. Of these phenotypes, the cerebral form usually manifests during early childhood with rapid cognitive and neurological deterioration and is accompanied by extensive white matter involvement. Adrenomyeloneuropathy, however, usually affects young adults and has focal symptoms typical of spinal cord and peripheral nerve involvement.

**Case presentation:**

A 35-year-old African American man with a history of alcohol abuse presented with personality changes and lower extremity weakness. Diffuse demyelination was found on the brain image, and a diagnosis of the cerebral form was made based on the clinical features and genetic test.

**Conclusions:**

We report a rare case of adult-onset cerebral X-linked leukodystrophy with a clinical phenotype of adrenomyeloneuropathy, and the diagnosis was confounded by a history of alcohol abuse.

## Background

X-linked adrenoleukodystrophy (X-ALD) is a peroxisome fatty acid beta-oxidation disorder that results in the accumulation of very long-chain fatty acids (VLCFAs) in tissues throughout the body; the highest levels of accumulation occur in myelin in the central nervous system, the adrenal cortex, and Leydig cells [1].

Clinically, X-ALD is a heterogeneous disorder caused by a mutation in the ATP-binding cassette transporter D1 (*ABCD1*) gene. The disease presents with several distinct phenotypes, with no clear genotype–phenotype correlation pattern. Adrenoleukodystrophy (ALD) is classified as the following: childhood cerebral phenotype (48%), adolescent cerebral phenotype (5%), adult cerebral phenotype (3%), adrenomyeloneuropathy (AMN) (25%), and isolated adrenal insufficiency (10%) [2].

Important factors for classification include brain magnetic resonance imaging (MRI), symptom onset time, and symptom progression. Cerebral X-ALD is usually suggested by cognitive deficits that occur in childhood, followed by rapid neurological and cognitive deterioration with progressive inflammatory cerebral demyelination. AMN is characterized by more focal symptoms without evidence of acute inflammation via brain MRI. We present a case of adult-onset cerebral X-ALD with the typical clinical features of AMN and confounded by a history of alcohol abuse.

## Case presentation

A 35-year-old African American man was initially sent to our emergency room to evaluate a possible head trauma after a witnessed fall. He complained of frequent falls and leg weakness for 3–4 months. The weakness appeared to be progressive and persistent without episodic worsening. He had to use a cane to compensate but was still able to ambulate. His family members also endorsed cognitive defects over the past few months before admission. These impairments consisted mainly of apathy and withdrawal from social interactions; he used to be fully independent but now stayed at home most of the time. His medical history was only significant for alcohol abuse (three to four cans of beer and red wine daily for 2 years), and his family history was unremarkable.

Physical examinations showed an age-appropriate, alert, and oriented man. He was able to engage in conversations with a paucity of speech and flat affect; he also made eye contact and followed all commands during our evaluations.

There was bilateral weakness with spasticity in all lower extremity muscle groups. The deep tendon reflex was hypoactive with an upward response of the plantar reflex on the left side. He also had a wide-based unsteady gait and poor limb coordination. The remainder of the neurological examination and a general examination were unremarkable.

**Fig. 1:**
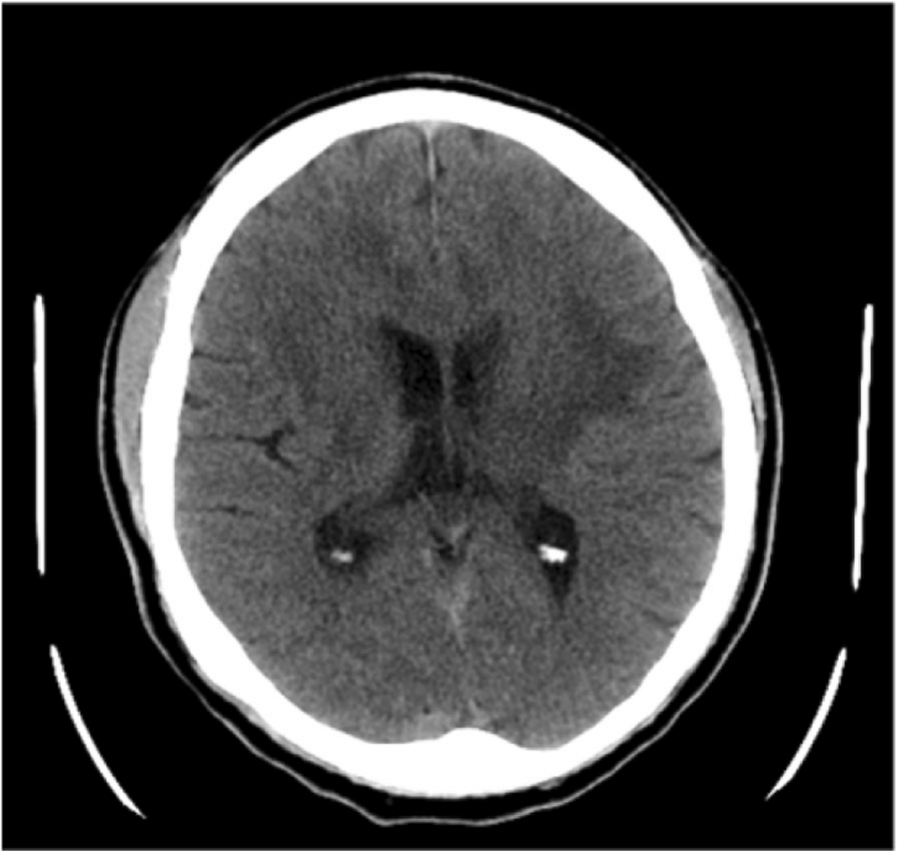
Computed tomography shows hypodense lesion within the left white matter and left basal ganglia

**Fig. 2:**
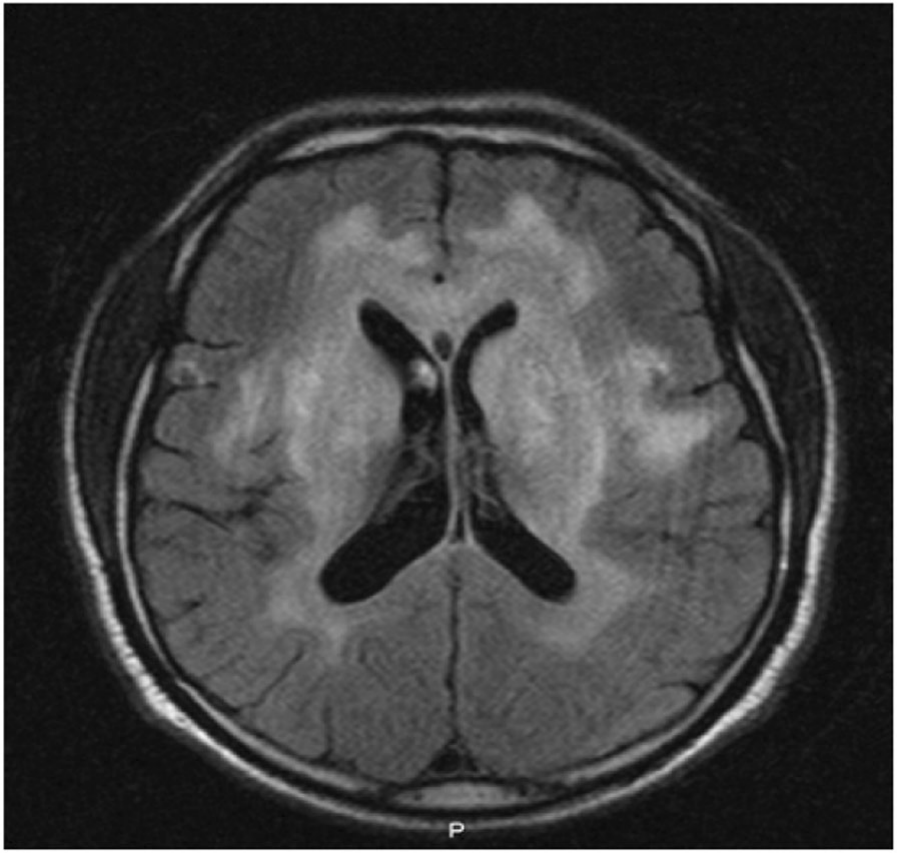
Magnetic resonance imaging shows confluent fluid-attenuated inversion recovery white matter signal involving supratentorial brain

**Fig. 3:**
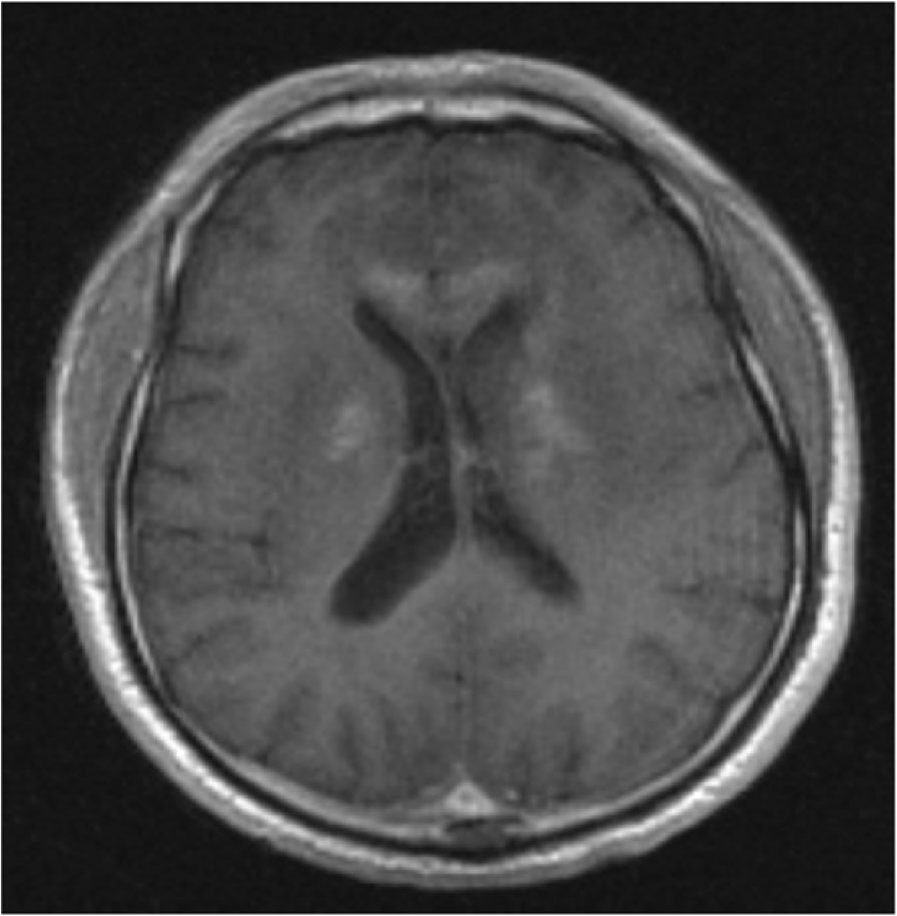
Magnetic resonance imaging shows bilateral enhancement involving corona radiata extending toward basal ganglia

Initial brain computed tomography (CT) in our emergency room demonstrated a mass lesion occupying the left caudate and extending into his frontal lobe (Fig. 1). A brain MRI showed bilateral and symmetric hyperintense signals in the corpus callosum, periventricular white matter, and internal capsule (Fig. 2) with an axial fluid-attenuated inversion recovery (FLAIR) sequence with gadolinium enhancement (Fig. 3). A cervical spine MRI was negative for cord compression and abnormal signals.

Routine blood tests, as well as human immunodeficiency virus (HIV), syphilis, and thyroid function tests, were unremarkable, and his serum vitamin B12 level was borderline (289 picogram per milliliter). A cerebral spinal fluid sample revealed a mildly elevated protein level (120 mg per deciliter) with a normal cell count (two cells per milliliter) and glucose level (79 mg per deciliter). The sample was negative for cytology, oligoclonal bands, culture, and Epstein–Barr virus DNA polymerase chain reaction (PCR). His urine toxicology panel was negative for common recreational drugs.

The initial impression was an acquired demyelinating white matter disease due to nutritional deficiency. A trial vitamin supplement (500 mg administered intravenously three times a day and 1000 μg of cyanocobalamin administered intramuscularly daily) was given based on his alcohol abuse history, but no symptom improvements were seen during the following week. An inherited condition was thus suspected given the negative workup, the non-response to treatment, and the symmetric involvement in the brain MRI. This suspicion was validated by his peroxisomal fatty acid profile (Table 1), and a diagnosis of X-ALD was confirmed according to the identification of a pathogenic mutation in the *ABCD1* gene: c.1489 2A>G (g.153005544). Adrenocortical insufficiency was not identified during the evaluation. No seizure activity was witnessed or shown on video-electroencephalography.

**Table 1:** Peroxisomal fatty acid profile

Test	Results	Unit	Reference value
C22:0	46.6	nmol/mL	≤ 96.3
C24:0	94.4	nmol/mL	≤ 91.4
C26:0	2.91	nmol/mL	≤ 1.30
C24:0/C22:0	2.02	Ratio	≤ 1.39
C26:0/C22:0	0.062	Ratio	≤ 0.023

*C22:0* docosanoic acid, *C24:0* tetracosanoic acid, *C26:0* hexacosanoic acid

His neurological condition deteriorated rapidly. He progressed into a non-verbal, quadriplegic patient dependent on a ventilator within 5 months of the initial encounter, and after 1 year of follow up he had degenerated into a vegetative state. Genetic counseling was offered to his family, and all of his siblings were negative for the *ABCD1* gene mutation.

## Discussion

AMN, the most common phenotype of ALD, usually manifests in male patients between 20 and 30 years of age and has an insidious progression. Weakness and spasticity of the lower extremities, sphincter dysfunction, and impotence are common symptoms. Neurologic dysfunction usually develops slowly over years, with only moderate increases in brain MRI signals of the white matter in the centrum ovale, internal capsule, or brainstem on FLAIR sequences without gadolinium enhancement; these disease characteristics suggest an intact blood–brain barrier and the absence of an active inflammatory process [3].

On the other hand, cerebral ALD is more severe and rare, with an age of onset of 5 to 12 years. Neurologic function is usually spared until demyelination is visible by MRI. Once the lesions become inflammatory, as evidenced by gadolinium enhancement on a T1 sequence, cognitive function deteriorates rapidly; common symptoms include frontal syndrome or new visual impairment, accompanied by motor deficits, ataxia, cortical blindness, or seizure. A vegetative state will ensue within 2 to 5 years [2, 4]. While 20% of patients with AMN develop the cerebral phenotype of X-ALD at a certain point of the disease course, symptoms of cerebral X-ALD followed by symptoms of AMN are exceedingly rare [2, 4, 5].

Elevated VLCFAs are the important biomarkers of X-ALD. Clinically three parameters are analyzed: the amount of hexacosanoic acid (C26:0), the ratio of tetracosanoic acid (C24:0)/docosanoic acid (C22:0), and C26:0/C22:0. The accumulation of VLCFA (in particular C26:0) is the result of the defective metabolism of peroxisomes and, consequently, exert a cytotoxic effect in neural cells. The neuropathological hallmark of AMN is an axonopathy, whereas neuroinflammation and cerebral demyelination are observed in cerebral ALD. Because of the absence of genotype–phenotype correlation in X-ALD, it is likely that a combination of genetic, epigenetic, and environmental factors play an essential role as triggers for the development of cerebral ALD [1].

This patient developed mild cognitive dysfunction, followed by lower extremity symptoms typical of AMN within months, and there was evidence of cerebral inflammatory demyelination. His psychomotor development during adolescence and early adulthood seemed uneventful, which is an uncommon feature of cerebral X-ALD. However, his cognitive impairment could have been overlooked for some time due to its subtle nature, and it was attributed initially to alcohol toxicity. On the other hand, the pronounced subacute neurological deficit characterized by lower extremity weakness with the brain MRI findings mimic acquired demyelinating diseases. This initial impression led to an extensive workup with negative findings. In such cases, detailed analyses of family history, personal history, and radiographic images with a high index of suspicion are crucial for diagnosis [6].

## Conclusions

We described an adult man with a history of alcohol abuse who presented with prominent clinical features of AMN. The symptoms later proved to be cerebral X-ALD based on *ABCD1* gene analysis, brain MRI findings, and rapid cognitive and neurological function deterioration. The clinical phenotypes of X-ALD can be diverse and overlap with each other. Therefore, this rare diagnosis should be entertained early to provide genetic counseling in a timely fashion.

## Abbreviations

*ABCD1*: ATP-binding cassette transporter D1
ALD: Adrenoleukodystrophy
AMN: Adrenomyeloneuropathy
C22:0: Docosanoic acid
C24:0: Tetracosanoic acid
C26:0: Hexacosanoic acid
CT: Computed tomography
FLAIR: Fluid-attenuated inversion recovery
HIV: Human immunodeficiency virus
MRI: Magnetic resonance imaging
PCR: Polymerase chain reaction
VLCFA: Very long-chain fatty acid
X-ALD: X-linked adrenoleukodystrophy
